# Mitral early-diastolic inflow peak velocity (E)-to-left atrial strain ratio as a novel index for predicting elevated left ventricular filling pressures in patients with preserved left ventricular ejection fraction

**DOI:** 10.1186/s12947-021-00248-z

**Published:** 2021-04-24

**Authors:** You Zhou, Cai-Ming Zhao, Zhen-Ya Shen, Xin Zhao, Bing-Yuan Zhou

**Affiliations:** First Affiliated Hospital of Soochow University, Suzhou, Jiangsu China

**Keywords:** Left atrial strain, Left ventricular diastolic dysfunction, Heart failure with preserved left ventricular ejection fraction

## Abstract

**Objectives:**

We sought to explore the relationship between an index of left ventricular diastolic function parameters combined with left atrial strain and the diastolic function of patients with preserved ejection fraction.

**Methods:**

We prospectively enrolled 388 patients with left ventricular ejection fraction (LVEF) ≥ 50%, 49 of whom underwent left heart catherization. Transthoracic echocardiography was performed within 12 h before or after the procedure. Left atrial (LA) strain was obtained by speckle tracking echocardiography. These patients served as the test group. The remaining patients (*n* = 339) were used to validate the diagnostic performance of the mitral early-diastolic inflow peak velocity (E)-to-left atrial reservoir strain ratio (E/LASr) in left ventricular diastolic dysfunction.

**Results:**

Invasive measurements of LV end-diastolic pressure (LVEDP) demonstrated that the E/LASr ratio was increased in patients with elevated LVEDP [ 2.0 (1.8–2.2) vs 3.0 (2.6–4.0), *p* < 0.001] in the test group (*n* = 49). After adjusting for age, mitral A, E/e' ratio and β-blocker use, the E/LASr ratio was an independent predictor of elevated LVEDP and showed good diagnostic performance in determining elevated LVEDP [area under the curve (AUC) 0.903, cutoff value 2.7, sensitivity 74.2%, specificity 94.4%]. In the validation group (*n* = 339), the E/LASr ratio also performed well in diagnosing elevated left atrial pressure (LAP) (AUC 0.904, cutoff value 3.2, sensitivity 76.5%, specificity 89.0%), while with a cut-off value of 2.7, the E/LASr ratio showed high accuracy in discriminating elevated LAP. In addition, E/LASr was a good index of excellent diagnostic utility (AUC: 0.899 to 0.996) in the categorization of diastolic dysfunction grades. Regarding the clinical relevance of this index, the E/LASr ratio could accurately diagnose HF with preserved ejection fraction (HFpEF) (0.781), especially in patients with “indeterminate” status (AUC: 0.829). Furthermore, an elevated E/LASr ratio was significantly associated with the risk of rehospitalization due to major adverse cardiac events (MACEs) within one year (odds ratio: 1.183, 95% confidence interval: 1.067, 1.312).

**Conclusions:**

In patients with EF preservation, the E/LASr ratio is a novel index for assessing elevated left ventricular filling pressure with high accuracy.

## Background

Over 90% of patients with heart failure (HF) have diastolic dysfunction regardless of left ventricular ejection fraction (LVEF), and left ventricular (LV) diastolic dysfunction is the predominant pathomechanism of HF with preserved ejection fraction (HFpEF) [1]. Impaired LV diastolic function will result in elevated LV filling pressure (LVFP), which is a major determinant of cardiac symptoms and prognosis in patients with chronic HF [2, 3]. Thus, the noninvasive estimation of LVFP obtained by echocardiography is important for diagnosing HFpEF and managing chronic HF.

The 2016 ASE/SCAI guideline employing several parameters makes it more convenient than previous versions; nonetheless, the diagnostic quandary of “indeterminate” status for patients whose data do not neatly fulfill the algorithms is still unsolved [4]. On the other hand, the accuracy of diagnostic diastolic dysfunction will decrease for patients with pulmonary arterial hypertension, low right atrial and right ventricular filling pressure, or severe tricuspid valve lesions [4]. Therefore, an accurate parameter for detecting LV diastolic function is needed.

Several studies have shown that left atrial (LA) strain, especially LA strain during the reservoir phase (LASr), is impaired in the setting of LV diastolic dysfunction and correlates well with LVFP or pulmonary capillary wedge pressure, suggesting that LASr is clinically useful for estimating LVFP [5, 6]. However, in some patients with coronary artery disease, LASr is not the best parameter for discriminating the filling pressure status [7]. Alternatively, combining LV and LA diastolic measurements could be more precise than a single parameter in predicting LVFP. In this regard, we conducted this study to explore the correlation of LVFP with the combination of LASr and LV diastolic measurements.

## Methods

### Population

We prospectively enrolled 394 patients treated at the First Affiliated Hospital of Soochow University from November 2018 to December 2019. Fifty-five of them who were suspected of having coronary artery disease or HFpEF underwent left heart catherization. LV end-diastolic pressure (LVEDP) was invasively measured by left heart catheterization. Standard transthoracic echocardiography was performed during the 12 h before or after the procedure, and LA strain was obtained by speckle tracking echocardiography. Six of the patients were excluded because echocardiographic imaging was not good enough; thus, 49 patients served in the test group. The remaining patients (*n* = 339) were used to validate the results of the test group and evaluate the diagnostic performance of E/LASr in left ventricular diastolic dysfunction.

The inclusion criteria were as follows: (1) LVEF ≥ 50%; (2) no severe valvular heart disease; and (3) presence of sinus rhythm. The exclusion criteria were as follows: (1) hemodynamic instability; (2) LVEF < 50%; (3) atrial fibrillation, atrial flutter, supraventricular tachycardia, or irregular ventricular rhythm; (4) severe heart valve disease: any mitral or aortic stenosis, moderate or greater tricuspid regurgitation, moderate or greater mitral regurgitation, or experience with any valvular heart surgery or interventions; (5) insufficient echocardiographic imaging; and (6) acute ST-segment elevation myocardial infarction or acute non-ST-segment elevation myocardial infarction.

### Conventional transthoracic echocardiography

Transthoracic echocardiographic measurements of all subjects were performed using a GE Vivid E9 or GE Vivid E95 (Norway) 2.5 MHz transducer in the left lateral decubitus position at rest. The biplane algorithm was used to measure the maximum volume of the left atrium in the standard apical four-chamber and two-chamber views before mitral valve opening for 1–2 frames. The LA maximal volume was divided by the body surface area to obtain the LA maximal volume index (LAVI). LVEF was measured in the standard apical four-chamber and two-chamber views by Simpson’s method biplane algorithm. Pulsed-wave Doppler (PW) was used to measure the peak early-diastolic (E) and peak end-diastolic (A) transmission velocity, E/A ratio, and E wave deceleration time at the level of the mitral leaflet tips from the apical four-chamber view. In the apical four-chamber view, the sampling points were placed at the levels of the basal portion of the septal and lateral mitral annulus. Tissue Doppler imaging (TDI) and PW were used to obtain the mitral annulus movement speed, and the peak value of the longitudinal movement in the early-diastolic period (i.e., septal e' and lateral e'). Then, the mean early-diastolic myocardial velocity (e′_mean_) and the ratio of E/e'_mean_ were calculated. The maximum velocity of tricuspid regurgitation (TR_max_) was measured by continuous-wave Doppler (CW) under the guidance of color Doppler from the parasternal long axis of the left ventricle or the apical four-chamber view. Researchers were blinded to the patient's LVEDP and clinical characteristics.

### Two-dimensional speckle tracking echocardiography

The left atrial strain was measured using the two-dimensional strain analysis package provided by the Echo PAC workstation (GE Healthcare). The two base points of the mitral annulus and the top of the distal end of the LA were manually selected; the area of interest was adjusted to include the entire LA wall, each view was divided into six sections, and twelve sections from each patient were analyzed. The global longitudinal LA strain was measured as an average of 12 sections. The LA reservoir strain was measured as the average of the longitudinal positive peak of LA strain, which was from all LA segments (i.e., 12 segments) of the apical 4-chamber and 2-chamber views [8].

### Invasive LV pressure measurements

The left ventricular filling pressure was measured using a 6F pigtail catheter. The invasive procedure was performed via the radial artery by an interventional cardiologist who was blinded to the echocardiography data. Before coronary angiography, transducers were balanced prior to the acquisition of hemodynamic data with zero level at the midaxillary line. After coronary angiography, left ventricular angiography was performed. The 6F pigtail catheter was reset routinely and placed in the left ventricle to obtain a stable pressure curve. Then, the ECG and left ventricular pressure curves were recorded simultaneously. Left ventricular end-diastolic pressure was measured at the QRS starting point for baseline stable left ventricular pressure curves. All parameters were averaged over three consecutive cardiac cycles. LVEDP > 16 mmHg was defined as elevated LVFP [1, 9].

### Diagnosis of left ventricular diastolic dysfunction

According to the recommendations of the 2016 ASE/SCAI guideline[4], 339 patients (the validation group) were assessed for left ventricular diastolic dysfunction. The following are the abnormalvalues of conventional LV diastolic parameters: (1) e' of TDI mitral annulus (septal e' < 7 cm/s or lateral e' < 10 cm/s), (2) E/e 'mean > 14, (3) LAVI > 34 ml/m^2^, and (4) TRmax > 2.8 m/s. When more than 50% of the above criteria were positive, the patients were diagnosed with LVDD, and LV diastolic function was considered normal when less than 50% of the above criteria were positive. In addition, when only 50% of the criteria were positive, patients were diagnosed as having indeterminate LV diastolic function.

### Definition of elevated left atrial pressure

According to the recommendations of the 2016 ASE/SCAI guideline [4], elevated left atrial pressure was defined as: mitral E/A ratio ≥ 2 or ≥ 2 positive criteria(LAVI > 34 mL/m^2^, E/e '_mean_ > 14, or TR_max_ > 2.8 m/s) when mitral E/A ratio ≤ 0.8 and E > 50 cm/s or mitral E/A ratio > 0.8 to < 2; and normal left atrial pressure was defined as: mitral E/A ratio ≤ 0.8 and E ≤ 50 cm/s or ≥ 2 negative criteria (LAVI > 34 mL/m^2^, E/e '_mean_ > 14, or TR_max_ > 2.8 m/s) when mitral E/A ratio ≤ 0.8 and E > 50 cm/s or mitral E/A ratio > 0.8 to < 2.

### Left ventricular diastolic dysfunction grade

According to the 2016 ASE/SCAI algorithm [4], the severity of patients with left ventricular diastolic dysfunction in the validation group was graded: when mitral E/A ratio ≤ 0.8, E ≤ 50 cm/s, and more than two of the three criteria ( E/e '_mean_ > 14, LAVI > 34 ml/m^2^, TR_max_ > 2.8 m/s) were negative, it suggested that the corresponding grade of diastolic dysfunction was grade I; when mitral E/A ratio ≥ 0.8 and E > 50 cm/s, or if the mitral E/A ratio was > 0.8 but < 2, and two or three of the three criteria were positive at the same time, it indicated that the corresponding grade of diastolic dysfunction was grade II; when mitral E/A ratio ≥ 2, it was diagnosed as grade III diastolic dysfunction [4].

### Diagnostic algorithm of HFpEF

According to the "HFA-PEFF diagnosis algorithm" offered by the 2019 ESC consensus recommendation [10], we performed clinical diagnosis of HFpEF on 339 patients in the validation group. The first was an initial workup, which included evaluating the symptoms and signs of heart failure and improving the clinical diagnosis of the primary disease (step 1). Then, the patients were assessed with echocardiography and natriuretic peptide. Diastolic function parameters of echocardiography and natriuretic peptide levels were used as the main basis for evaluating HFpEF. Then the patients were scored according to the scoring system (shown in Fig. 3 of the "HFA-PEFF Diagnosis Algorithm" [10]) (step 2). A score ≥ 5 points implied definite HFpEF. An intermediate score (2–4 points) implied diagnostic uncertainty and further hemodynamic testing was recommended, including echocardiography or invasive hemodynamic exercise stress testing (step 3). Symptoms compatible with HF could be confirmed to originate from the heart if hemodynamic abnormalities were detected either at rest or during exercise.

### Definition of MACEs

Major adverse cardiac events (MACEs) included all-cause mortality, acute myocardial infarction, HF, stroke, and coronary revascularization.

### Statistical analysis

Statistical analysis was performed using SPSS version 25.0 software. Continuous variables that were normally distributed are presented as the mean ± SD and were analyzed with an independent t-test. Variables that were not normally distributed are presented as medians with interquartile ranges (IQR = 25th–75th percentile) and were analyzed with the Mann–Whitney U test. Categorical data are expressed as absolute numbers or percentages and were analyzed with the chi-squared test. Univariate logistic regression was used to calculate odds ratios to predict LVEDP. All variables with *p* ≤ 0.100 (including LASr, E/LASr, peak A, E/e'_mean_, β-blockers) and age were included in the multiple logistic regression analysis to explore the relevance of LVEDP. *P* < 0.05 (two-tailed) was considered statistically significant. In our four models, LASr and E/LASr were analyzed separately due to their multicollinearity, with other control variables kept the same. The C-statistic was calculated in each model to allow comparison between them. In the test group, the area under the curve (AUC) of the receiver operating characteristic curve was used to compare the performance of multiple variables in determining elevated LVEDP. In the validation group, receiver operating characteristic curve analysis was used to evaluate the accuracy of the E/LASr ratio for diagnosing left ventricular diastolic dysfunction, grading the severity of LVDD and HFpEF. Univariate logistic regression was used to analyze the correlation between different variables and rehospitalization due to MACEs within one year.

## Results

### Characteristics of the study population

The study finally included 388 patients, including 49 in the test group and 339 in the validation group. The test group that underwent left heart catheterization was divided into a normal LVEDP group (*n* = 18) and an elevated LVEDP group (*n* = 31) according to whether the LVEDP was greater than 16 mmHg. There were no significant differences in sex, age, medical history, coronary angiography results or other conventional echocardiographic indicators, such as LVEF, LAEF, and left ventricular diastolic function indicators, between the two groups. Compared with the patients in the normal LVEDP group, those in the elevated LVEDP group showed significantly lower LASr (32.9 ± 1.5 vs 23.2 ± 1.2, *p* < 0.001), and E/LASr was significantly increased [ 2.0 (1.8–2.2)vs 3.0 (2.6–4.0), *p* < 0.001] (Table 1).

**Table 1:** Characteristics of patients in two groups

Variable	LVEDP ≤ 16 mmHg (*n* = 18)	LVEDP > 16 mmHg (*n* = 31)	*P*	validation group (*n* = 339)
Baseline Characteristics				
Female (n (%))	4 (22%)	11 (30.6%)	0.332	172 (50.7%)
Age(year)	64 ± 2	63 ± 2	0.563	63 ± 1
HR (beats/min)	63 ± 2	68 ± 2	0.144	72 ± 1
BMI(kg/m^2^)	24.1 ± 0.7	24.6 ± 0.5	0.641	24.1 ± 0.2
Medical history, n (%)				
Hypertension	11 (61%)	26 (83%)	0.149	198 (58.4%)
CAD	9 (45%)	11 (31%)	0.314	77 (22.7%)
CKD(stage ≥ 3)or ESRD	1 (6%)	1 (3%)	1.000	24 (7.1%)
COPD	1 (6%)	1 (3%)	1.000	8 (2.4%)
Diabetes	3 (17%)	8 (25.8%)	0.701	70 (20.6%)
Medication, n (%)				
β-Blockers	5 (28%)	17 (55%)	0.066	——
ACEI	5 (28%)	8 (26%)	0.880	——
ARBs	3(17%)	11 (35.5%)	0.160	——
Calcium blocker	5 (28%)	14 (45%)	0.368	——
Diuretics	2 (11%)	3 (10%)	0.873	——
Statins	18 (100%)	30 (97%)	1.000	——
Nitrates	5 (28%)	8 (26%)	1.000	——
Antiplatelet drugs	20 (100%)	35 (100%)	1.000	——
Angiographic findings				——
Numbers of vessels with significant stenosis > 50%				——
1	3	6	1.000	——
2	2	2	0.974	——
3	2	2	0.623	——
Vessels with stenosis > 50%				——
LM	1	2	1.000	——
LAD	5	6	0.744	——
LCX	5	2	1.000	——
RCA	1	3	1.000	——
Echocardiographic Variables				
Mitral E (cm/s)	66 ± 2	71 ± 2	0.143	79 ± 1
Mitral A (cm/s)	75 ± 4	88 ± 4	0.045	86 ± 1
E/A ratio	0.8 (0.7—1.0)	0.8 (0.7—0.9)	0.383	0.9 (0.8—1.1)
Septal e′ velocity (cm/s)	6.8 ± 0.5	6.3 ± 0.3	0.363	6.4 ± 0.1
Lateral e′ velocity (cm/s)	10 (7.8—12.0)	8.0 (6.0—10.4)	0.144	9(6—11)
E/e’_mean_ ratio	8.0 (6.8—10.6)	10 (8.2—11.2)	0.078	10 (8—15)
TR peak velocity (m/s)	2.3 (2.1—2.5)	2.2 (2.1—2.6)	0.949	2.5 (2.3—2.8)
LVEF (%)	66 ± 2	64 ± 1	0.733	64 ± 1
LAVimax (ml/m2)	14.6 ± 1.7	16.5 ± 1.2	0.388	32.5 ± 0.7
LAEF (%)	55 ± 2	55 ± 2	0.851	54 ± 1
LASr(%)	32.9 ± 1.5	23.2 ± 1.2	< 0.001	27.2 ± 0.5
E/LASr ratio	2.0 (1.8—2.2)	3.0 (2.6—4.0)	< 0.001	2.7 (2.1—3.8)

### Logistic regression analysis and prediction model

Four different multivariate logistic regression analyses showed that after adjusting for age, peak A, E/e'_mean_ ratio and other factors, LASr and E/LASr were independent predictors of LVEDP > 16 mmHg in their respective models. The models showed that E/LASr has a higher C-statistic than the model with LASr (Table 2).

**Table 2:** Multivariate regression analysis to identify predictors of elevated LVEDP

Model	Variables	Univariate analysis		Multivariate analysis		C-statistic
		OR (95% CI)	*P*	OR (95% CI)	*P*	
1	LASr	0.817 (0.730,0.915)	< 0.001	0.766 (0.655,0.896)	0.001	0.934
	Age	0.982 (0.926,1.042)	0.555	0.885 (0.781,1.004)	0.058	
	Mitral A	1.032 (1.000,1.065)	0.053	1.059 (0.997,1.124)	0.063	
	E/e’_mean_ ratio	1.236 (0.971,1.572)	0.085	1.093 (0.799,1.496)	0.577	
	β-Blockers	0.317 (0.091,1.106)	0.072	0.120 (0.015,0.962)	0.046	
2	E/LASr	21.516 (3.527,131.264)	0.001	85.720 (4.533,1621.029)	0.003	0.946
	Age	0.982 (0.926,1.042)	0.555	0.926 (0.826,1.038)	0.185	
	Mitral A	1.032 (1.000,1.065)	0.053	1.076 (0.999,1.159)	0.052	
	E/e’_mean_ ratio	1.236 (0.971,1.572)	0.085	0.798 (0.547,1.165)	0.243	
	β-Blockers	0.317 (0.091,1.106)	0.072	0.069 (0.007,0.706)	0.024	

### The accuracy of LVEDP > 16 mmHg predicted by LASr and its combination index

**Fig. 1:**
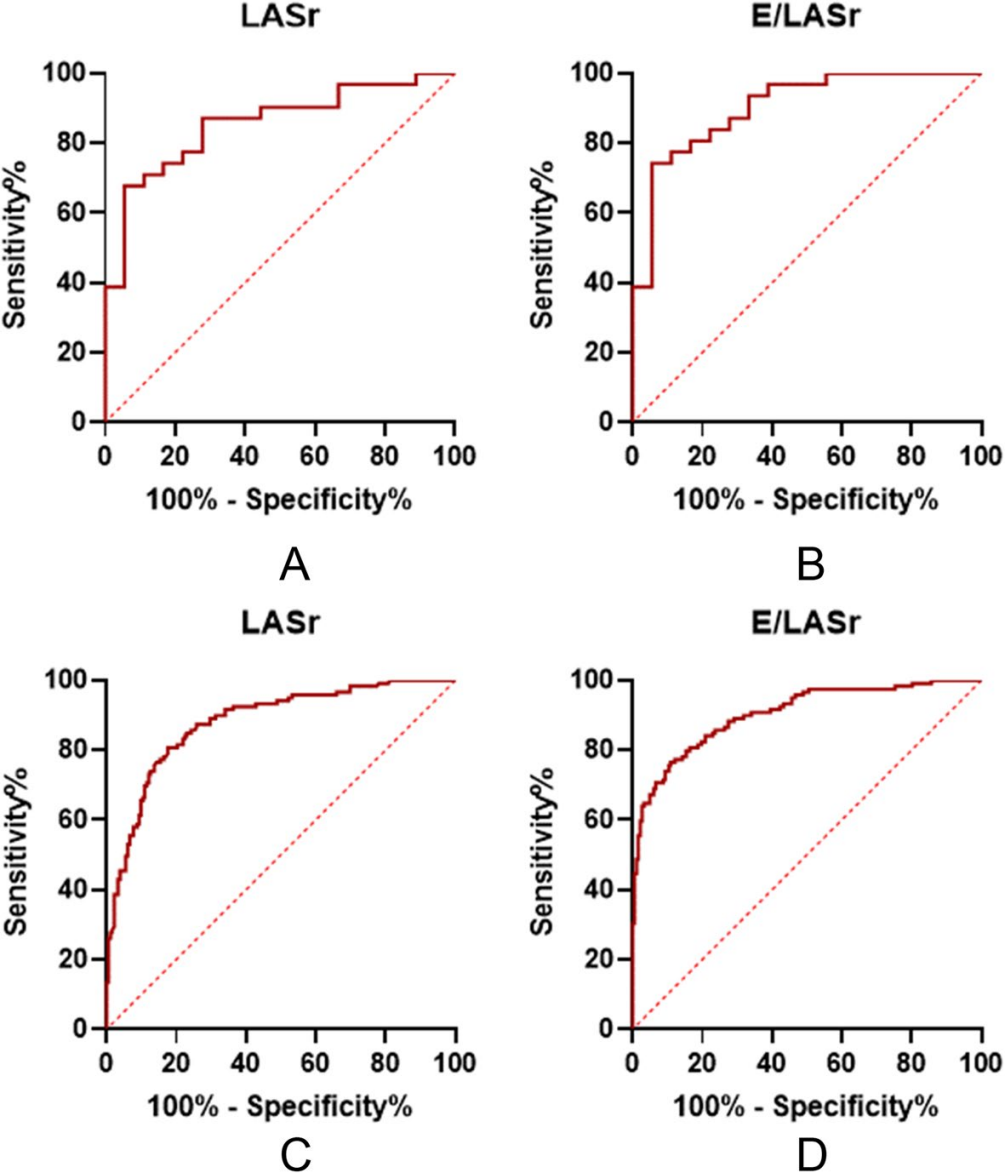
**a**: ROC curves of LASr for the prediction of LV filling pressure in the test group; **b**: ROC curves of E/LASr for the prediction of LV filling pressure in the test group; **c**: ROC curves of LASr for the prediction of LAP in the validation group; **d**: ROC curves of E/LASr for the prediction of LAP in the validation group

LASr and E/LASr (AUC 0.903, cutoff value 2.7, sensitivity 74.2%, specificity 94.4%) had good diagnostic accuracy for elevated LVEDP. The diagnostic performance of E/LASr for LVEDP > 16 mmHg is better than that of LASr (Fig. 1 and Table 3).

**Table 3:** The performance of LASr and E/LASr for determining elevated LVEDP or LAP

	cut-off value	AUC	Sensitivity	Specificity	Accuracy
Test group					
LASr(%)	24.7	0.855	94.4%	67.7%	77.5%
E/LASr	2.7	0.903	74.2%	94.4%	83.7%
Validation group					
LASr(%)	24.8	0.880	82.4%	80.7%	66.1%
E/LASr	3.2	0.904	76.5%	89.0%	96.1%
E/LASr	2.7		85.7%	74.2%	77.1%

Among the 339 patients in the validation group, 119 patients were diagnosed with elevated LAP according to the 2016 ASE/SCAI guideline. In agreement with these findings in the test group, E/LASr had good accuracy in diagnosing elevated LAP (Fig. 1 and Table 3).

### E/LASr ratio and left ventricular diastolic function classification

**Fig. 2:**
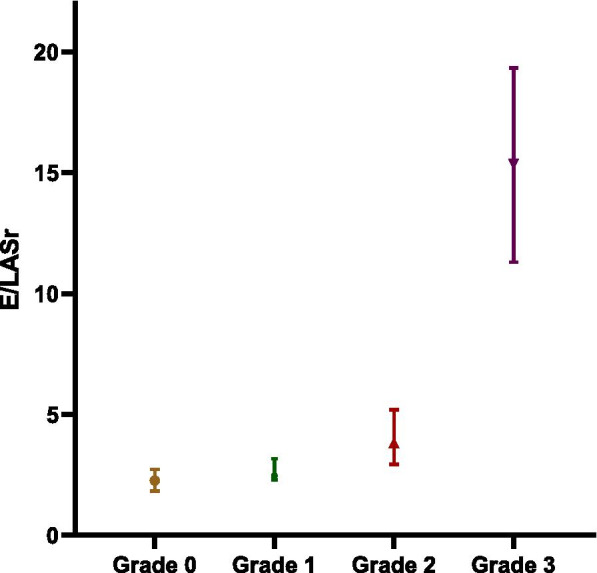
E/LASr value(IQR) according to diastolic function classification, Grade 0 vs Grade 1 *P* = 0.033; Grade 1 vs Grade 2 *P* < 0.001; Grade 2 vs Grade 3 *P* < 0.001. Grade 0, normal diastolic function; Grade 1, diastolic dysfunction grade I; Grade 2, diastolic dysfunction grade II; Grade 3, diastolic dysfunction grade III

According to the 2016 ASE/SCAI guideline, patients in the validation group (*n* = 339) were divided into normal diastolic function (grade 0, *n* = 183), diastolic dysfunction grade I (*n* = 9), diastolic dysfunction grade II (*n* = 101) and diastolic dysfunction grade III (*n* = 8). There were significant differences in E/LASr among the groups (Fig. 2). The E/LASr ratio had higher sensitivity and specificity in evaluating the severity of LVDD, and as diastolic dysfunction worsened, its accuracy was better (Table 4).

**Table 4:** Cutoff value and accuracy of left ventricular diastolic dysfunction grading evaluated by E/LASr

	E/LASr Cut-off value	AUC	Sensitivity	Specifiety	Accuracy
Grade 0 vs grade 1–3	3.2	0.899	76.3%	88.5%	83.1%
Grade 0–1 vs grade 2–3	3.3	0.923	78.9%	90.1%	87.8%
Grade 0–2 vs grade 3	8.6	0.996	100.0%	98.3%	98.3%

### E/LASr ratio and HFpEF

Among the 339 patients in the validation group, 37 were clinically diagnosed with HFpEF. There was a significant difference in E/ LASR between the two groups (Table 5).

**Table 5:** Characteristics of HFpEF and non-HF patients in the validation group

Variable	HFpEF (*n* = 37)	non-HF (*n* = 302)	*P*
Female (n (%))	25(68%)	147(49%)	0.036
Age (year)	68 ± 2	62 ± 1	0.013
HR (beats/min)	75 ± 2	70 ± 1	0.150
BMI (kg/m^2^)	24.4 ± 0.7	24.1 ± 0.2	0.828
Hypertension	29(78%)	169(56%)	0.012
CAD	9(24%)	68(22%)	0.836
CKD(stage ≥ 3)or ESRD	8(22%)	16(5%)	0.002
COPD	1(3%)	7(2%)	1.000
Diabetes	10(27%)	60(20%)	0.291
Mitral E (cm/s)	96 ± 4	77 ± 1	< 0.001
Mitral A (cm/s)	82 ± 6	87 ± 1	0.249
E/A ratio	0.9(0.8—2.0)	0.9(0.7—1.1)	0.014
Septal e′ velocity (cm/s)	5.0 ± 0.2	6.6 ± 0.1	< 0.001
Lateral e′ velocity (cm/s)	6.0(5.0—8)	9.0(7.0—11.0)	< 0.001
E/e’ ratio	16.2(14.1—16.2)	9.9(7.4—14.6)	< 0.001
TR peak velocity (m/s)	2.8(2.7—3.0)	2.5(2.3—2.7)	< 0.001
LVEF (%)	60 ± 1	65 ± 1	0.001
LAVimax (ml/m2)	42.8 ± 2.0	31.2 ± 0.7	< 0.001
LAEF (%)	45 ± 3	55 ± 1	< 0.001
LASr(%)	18.0 ± 1.5	28 ± 1	< 0.001
E/LASr ratio	4.8(3.2—9.1)	2.6(2.0—3.6)	< 0.001

**Fig. 3:**
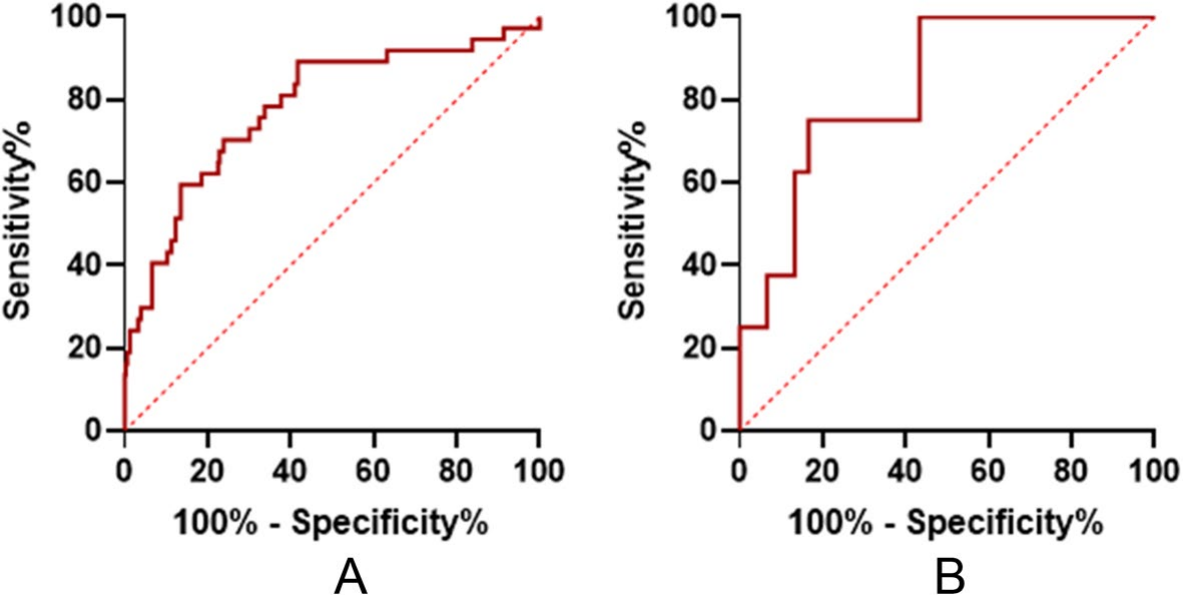
**a**: ROC curves of E/LASr for the prediction of HFpEF in the validation group, AUC: 0.781, cutoff value 2.9, sensitivity 89.2%, specificity 58.3%; **b**: ROC curves of E/LASr for the prediction of HFpEF in indeterminate status patients, AUC: 0.829, cutoff value 3.6, sensitivity 75.0%, specificity 83.4%

ROC curve analysis suggested that E/LASr performed well in diagnosing HFpEF. In the validation group, when diastolic function was assessed according to the 2016 ASE/SCAI guideline algorithm, 38 people were classified as having indeterminate diastolic function, and 7 of them were clinically diagnosed with HFpEF. E/LASr was used to discriminate HFpEF with high accuracy in patients with indeterminate diastolic function, which suggested that E/LASr may be used for the diagnosis of heart failure in the gray area with indeterminate diastolic function (Fig. 3).

### Correlation between the E/LASr ratio and rehospitalization due to MACEs within one year

**Fig. 4:**
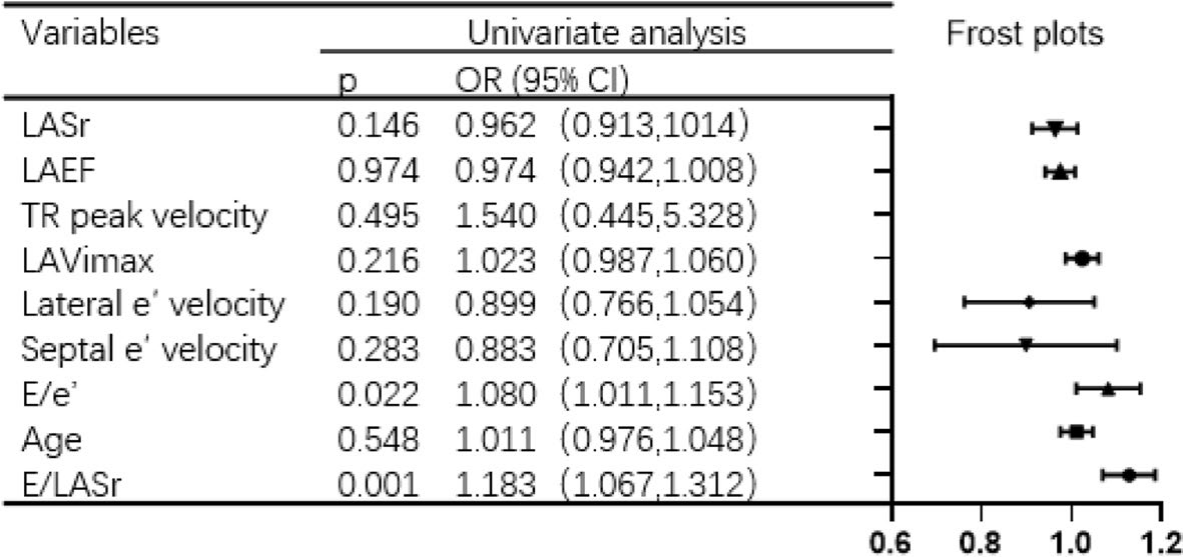
Correlation between related parameters of diastolic function and MACEs

Within one year, 18 people in the validation group were hospitalized for treatment due to MACEs. Univariate logistic regression analysis showed that patients with an elevated E/LASr ratio had an increased risk of MACEs [OR: 1.183, 95% CI: (1.067, 1.312), *p* = 0.001], while other traditional diastolic function parameters were poorly correlated with MACEs (Fig. 4).

## Discussion

This study aimed to evaluate the predictive value and potential clinical relevance of a new combination index, E/LASr, for elevated left ventricular filling pressure in patients with normal LVEF. We found that LASr and E/LASr were both independent predictors of elevated LVEDP. More importantly, we found that the combined E/LASr index predicts elevated LVEDP or LAP with increased accuracy. In addition, E/LASr can accurately diagnose HFpEF, particularly in patients with an “indeterminate” status. Furthermore, an elevated E/LASr ratio was significantly associated with the risk of rehospitalization due to MACEs within one year.

Recently, LA function measured as LA reservoir strain (LASr) has been shown to be significantly related to invasive left ventricular filling pressure [5, 6, 11]. This was also confirmed in our study [5].

Left atrium reservoir function reflects the relaxation and compliance of the left atrium and is regulated by left ventricular systolic function [12]. The left atrium is directly exposed to left ventricular pressure during diastolic mitral valve opening. In the early stages of left ventricular diastolic dysfunction, the left atrium can still contract to compensate for the elevated left ventricular pressure. However, in the case of long-term high left ventricular pressure, compliance of the left atrium gradually becomes blunt, resulting in a decrease in the reserve of the left atrium, which ultimately leads to enlargement and failure of the left atrium [13]. In fact, in the case of elevated left ventricular pressure, even if the left atrium has not yet expanded, the function of the left atrium has been impaired [14]. Therefore, LASr can reflect elevated left ventricular filling pressure in the early stage.

This study found that combining the early-diastolic peak inflow velocity (E) of the mitral valve and left atrial reservoir strain (LASr) as a single index further improved the ability to discriminate elevated left ventricular filling pressure (AUC = 0.903) in patients with preserved LVEF with higher accuracy. The combination index E/LASr was composed of the patient’s current left ventricular filling state (mitral valve E velocity) and its LA function change (LASr). This reflected not only the influence of the pressure gradient between the LA and LV on LV filling but also the relaxation and compliance of the LA affected by LV diastolic function. Therefore, this index was a more comprehensive indicator for prediction of elevated left ventricular filling pressure.

This study further verified the accuracy of the E/LASr ratio in evaluating left ventricular diastolic dysfunction in 339 patients with LVEF ≥ 50% in the validation group. However, the invasive LVEDP cutoff value (2.7) predicted by the E/LASr ratio in the test group was lower than the elevated LAP cutoff value (3.2) for the evaluation of the E/LASr ratio in the validation group. Previous studies have shown that in the early stages of left ventricular diastolic dysfunction, only LVEDP is elevated, while LA pressure and mean pulmonary capillary wedge pressure are still normal [4, 15]. However, the algorithm of the 2016 ASE/EACVI guideline is based on the prediction of mean pulmonary capillary wedge pressure, not LVEDP [4, 15]. In addition, traditional diastolic function parameters such as LAVI used in the 2016 ASE/EACVI guideline algorithm have been used as chronic and severe surrogates for left ventricular diastolic dysfunction, but LAVI is an insensitive biomarker in the early stages of diastolic dysfunction [16]. This may lead to the diastolic dysfunction identified in the validation group according to the 2016 guidelines no longer being limited to the early stage. To a certain extent, these reasons may explain why the cutoff value of E/LASr in the test group was lower than that in the verification group. We also proved that the E/LASr ratio can grade the severity of left ventricular diastolic function with good accuracy. In addition, as diastolic function worsened, the accuracy of its classification was better.

Regarding the clinical relevance of E/LASr, this study found that E/LASr can accurately diagnose HFpEF in the validation group, and even among patients classified as "indeterminate diastolic function", it can accurately distinguish patients with HFpEF. This shows that the E/LASr ratio added the value of supplementary diagnosis to the 2016 guidelines, especially for the diagnosis of HF in gray areas with indeterminate diastolic function. In addition, in this study, patients with an elevated E/LASr ratio had a significantly increased risk of MACEs. To a certain extent, this is consistent with the results of some recent studies. Braunauer et al. found that an elevated E/LASr ratio was significantly associated with worse functional capacity and HF hospitalization at 2 years [17]. A study of patients with atrial fibrillation found that an elevated E/LA strain ratio was associated with HF hospitalizations or worse cardiovascular events [18]. A study in hemodialysis patients found that the E/LA strain ratio is a useful parameter for predicting the total mortality and cardiovascular mortality of hemodialysis patients [19]. Further prospective studies are warranted to validate these findings.

### Limitations

This study has several limitations. First, the sample size for invasive measurement of left ventricular filling pressure is limited, and it is necessary to increase the sample size for multicenter studies to further verify our results. Second, the left atrial strain measured by speckle tracking imaging is defined as the absolute strain value of the three phases of the left atrium, and this study measured and analyzed only the left atrial reservoir strain. Third, because patients with atrial fibrillation lack effective atrial contraction and patients with severe mitral stenosis or mitral regurgitation have an abnormally enlarged left atrium, we did not include such patients. Finally, the clarity of echocardiographic images affects the repeatability and credibility of the left atrial strain results. Therefore, the acquisition of left atrial images and analysis of strains require more skilled operators. However, an increasing number of studies have confirmed that left atrial strain may be a powerful indicator for evaluating left ventricular diastolic function, which makes it possible for left atrial strain to be included in the diagnosis and classification of left ventricular diastolic dysfunction in the future.

## Conclusion

The results of this study indicate that a novel combination index (E/LASr) may be a more accurate indicator in predicting elevated LVFP and assessing diastolic dysfunction in patients with preserved EF. This indicator can not only add complementary diagnostic value to the 2016 ASE/SCAI guideline but also has potential clinical relevance for adverse cardiovascular events, which is worthy of further study.

## Data Availability

The datasets during and/or analysed during the current study available from the corresponding author on reasonable request.
